# Sickle Cell Disease and Gut Health: The Influence of Intestinal Parasites and the Microbiome on Angolan Children

**DOI:** 10.3390/ijms25137258

**Published:** 2024-07-01

**Authors:** Mariana Delgadinho, Catarina Ginete, Brígida Santos, Jocelyne Neto de Vasconcelos, Ana Paula Arez, Miguel Brito

**Affiliations:** 1 H&TRC-Health & Technology Research Center, ESTeSL-Escola Superior de Tecnologia da Saúde, Instituto Politécnico de Lisboa, 1990-096 Lisbon, Portugal; mariana.delgadinho@estesl.ipl.pt (M.D.); catarina.ginete@estesl.ipl.pt (C.G.); 2 Centro de Investigação em Saúde de Angola (CISA), Caxito, Angola; santosbrigida@yahoo.com.br (B.S.); jocelyne.vasconcelos@gmail.com (J.N.d.V.); 3 Instituto Hematológico Pediátrico, Luanda, Angola; 4 Global Health and Tropical Medicine (GHTM), Associate Laboratory in Translation and Innovation towards Global Health, (LA-REAL), Instituto de Higiene e Medicina Tropical (IHMT), Universidade NOVA de Lisboa (UNL), 1099-085 Lisbon, Portugal; aparez@ihmt.unl.pt

**Keywords:** Parasitic infections, gut microbiome, sickle cell disease, *Ascaris lumbricoides*, *Giardia intestinalis*, *Lactobacillus*, *Bifidobacterium*

## Abstract

Parasitic infections are a common problem in developing countries and can intensify morbidity in patients with sickle cell disease (SCD), increasing the severity of anemia and the need for transfusions. It has been demonstrated that both helminths and protozoa can affect gut microbiome composition. On the other hand, the presence of specific bacterial communities can also influence parasite establishment. Considering this, our aim was to associate the presence of intestinal parasites with the results of hematological analyses and microbiome composition evaluations in a population of Angolan children with and without SCD. A total of 113 stool samples were collected, and gut microbiome analysis was performed using 16S sequencing and real-time PCR to detect eight different intestinal parasites. In our population, more than half of children (55%) had at least one parasitic infection, and of these, 43% were co-infected. *Giardia intestinalis* and *Ascaris lumbricoides* were more frequently found in children from the rural area of Bengo. Moreover, SCD children with ascariasis exhibited higher values of leukocytes and neutrophils, whereas the total hemoglobin levels were lower. In regards to the gut microbiome, the presence of intestinal parasites lowered the prevalence of some beneficial bacteria, namely: *Lactobacillus*, *Bifidobacterium*, *Cuneatibacter*, *Bacteroides uniformis*, *Roseburia*, and *Shuttleworthia*. This study presents the prevalence of several intestinal parasites in a high-risk transmission area with scarce information and opens new perspectives for understanding the interaction between parasites, the microbiome, and SCD.

## 1. Introduction

Sickle cell disease (SCD) is a common genetic disease and a growing health problem in many parts of the world, affecting about 300,000 newborns every year [1,2]. It is considered a lifelong disease characterized by chronic hemolytic anemia, unpredictably painful episodes, and widespread organ damage that leads to premature death [3]. Sickle erythrocytes can often lead to vaso-occlusive crisis (VOC), which represent one of the most variable and common complications in this disease in terms of intensity, chronicity, or frequency [3,4]. Hydroxyurea (HU) is the leading treatment for these patients, since it significantly reduces VOC frequency, organ damage, acute chest syndrome, and the need for blood transfusions [5]. However, among SCD patients, there is substantial phenotypic heterogeneity which supports the idea that several factors play an essential role in disease modulation [6]. In Sub-Saharan Africa, where 80% of SCD children are born, between 50% to 90% die before turning five [1,7]. Most of these children die from infections [3], as SCD substantially increases the risk for various types of infections (bacterial, viral, and parasitic) in early life [8]. These infections become more severe during VOC occurrences and severe anemia [8]. SCD children manifest several immunologic abnormalities, including decreased splenic function, diminished opsonization capability, and abnormal neutrophil function, which compromises their innate immune response and increases the risk of sepsis, eventually leading to the high mortality rate associated with this disease [7,8].

The presence of intestinal parasites, such as *Giardia intestinalis*, *Entamoeba histolytica*, *Entamoeba coli*, *Ascaris lumbricoides*, *Ancylostoma duodenale*, *Trichuris trichiura*, and *Strongyloides stercoralis*, may worsen SCD symptoms, causing malabsorption, nutritional deficiencies, growth retardation, and severe anemia [8,9]. About 3.5 billion people worldwide are affected by intestinal parasitic infections, and the majority of these are children [9,10]. A high prevalence of intestinal parasites is particularly found in regions of low socioeconomic status, such as overcrowded living areas with unsafe water sources, poor environmental sanitation, improper waste disposal, or unhygienic habits [9]. 

In a study involving 791 fecal samples from children aged 6 to 10 years in Bie Province (Angola), it was reported that 80% of the samples were infected with intestinal helminths, intestinal protozoa, or a combination of both. The most prevalent included *A. lumbricoides*, *T. trichiura*, *Entamoeba coli*, *G. intestinalis*, hookworm, and *Hymenolepis nana* [11]. In the western part of Angola (Cubal), there was a high prevalence of parasites, i.e., *S. stercoralis*, *G. intestinalis*, and *Blastocystis* spp. (21.4%, 37.9%, and 25.6%, respectively) in school-aged children [12]. As for SCD patients, a study in Nigeria revealed that the positive cases inlcuded four main classes of parasites, including: nematodes (*A. lumbricoides* and *Hookworm*), trematodes (*Schistosoma mansoni*), cestodes (*H. nana*), and protozoans (*E. histolytica*) [13] The prevalence and burden of parasites can vary greatly between countries but also between provinces. Therefore, studies on the prevalence of various intestinal parasitic infections are a necessity, not only to identify the cause of the outbreak, but also to implement effective control strategies, such as deworming and public health campaigns, in order to treat and prevent further cases among the affected community. 

The gut microbiota is considered a complex ecosystem essential for human health, encompassing bacteria, viruses, fungi, helminths, and protozoa which interact and compete with each other [14]. Indeed, parasites and bacteria have co-evolved along with the intestinal immune system, resulting in a complex interaction network within the human mucosa [15,16]. Research studies have shown that this parasite–microbiota interaction might determine infection symptomatology, induction of dysbiosis, alteration of parasite virulence, and overall clinical outcomes inside the host [17,18]. Parasites can not only dramatically shape the physical and immune landscape of the gut microbiome and change host interactions with its bacterial flora, either driving or protecting against dysbiosis and inflammatory processes [16], but the presence of specific bacterial communities can also influence parasite establishment by altering the colonization success, replication, or virulence [16,19]. Thus, the balance between the host and gut microbiome is disturbed once parasites colonize the gut, especially when both groups are competing for the same resources [19]. 

Helminth parasites can lead to bacterial translocation in the host, which could cause sepsis [20]. However, there are studies reporting that helminth infections can also increase bacterial diversity and downregulate inflammation [20,21]. As for protozoa, they are known to alter the diversity of the microbiome during colonization of the intestine, which can alter mucosal immune homeostasis and promote diseases [22]. For example, *Giardia* and *Cryptosporidium* promote a shift in microbial community structure associated with dysbiosis, which facilitates bacterial translocation [22]. However, the precise parasite factors and how they affect bacterial composition in certain diseases remain largely understudied.

The disruption of intestinal mucosal barriers, a critical player in gut homeostasis maintenance, is also potentiated in SCD due to, among other factors, the recurrent VOC, activated neutrophils, and therapeutic antibiotic cycles, which might promote bacterial translocation and subsequent bacteremia episodes in these highly susceptible patients [23]. In fact, recent studies have previously demonstrated the presence of pathophysiological and microbial changes in the gut of SCD patients, including enterocyte injury, increased permeability, altered microbial composition, and bacterial overgrowth [24,25]. Our research group has also contributed to this area by reporting a lower abundance of *Blautia* and a higher prevalence of the genus *Clostridium XI* in SCD children compared to healthy controls [26]. Moreover, we reported that these children, after hydroxyurea treatment, expressed higher proportions of several beneficial bacteria, mostly short-chain fatty acids-producing species [27]. As an extension of these previous studies, in this work, we use real-time PCR to detect specific intestinal parasites known to be more prevalent in Angola, especially in Luanda and Bengo, where this population resides, since the presence of these parasites could be modulating the gut microbiome, affecting the immunological system of the individual, and consequently altering the risk of developing a more severe SCD phenotype.

## 2. Results

### 2.1. Prevalence of Parasitic Infections

**Figure 1:**
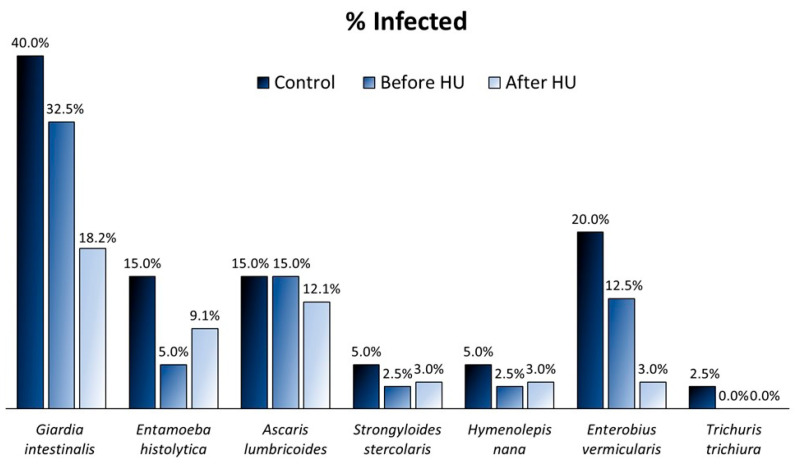
Prevalence of infection in an Angolan pediatric population in the three groups of study: controls without the SS genotype (*n* = 40), SCD children before HU treatment (*n* = 40), and SCD children after 6 months of HU treatment (*n* = 33).

In the 80 samples, the total prevalence of *G. intestinalis* was 36.3%, of *E. vermicularis*, 16.3%; of *A. lumbricoides*, 15.0%; of *E. histolytica*, 10.0%; of *S. stercoralis*, 3.8%; of *H. nana*, 3.8%; and of *T. trichiura*, 1.3%. Figure 1 represents the prevalence of infected/positives for each parasite in the different population groups. There were no positive cases for *S. mansoni.* In our population, more than half of the children had at least one parasitic infection (55.0%) and of these, 43.2% were co-infected. Moreover, 60.7% of the SCD children shared the infection with their respective sibling(s).

The control individuals presented the highest infection prevalence (60.0%) compared to the SCD children before HU (50.0%) or after 6 months of HU treatment (36.4%). However, infection prevalence of each parasite in the controls and SCD before HU group, which expressed the same sample collection data, did not significantly differ (Chi-square test). Additionally, after the hydroxyurea treatment, there was a reduction of 44.1% in SCD *Giardia*-positives, 19.2% in *Ascaris*-positives, 75.8% in *Enterobius*-positives, and an increase of 81.8% in *Entamoeba*-positives, but none of these decreases were statistically significant (Fisher’s exact test).

### 2.2. Parasite Prevalence according to Residency

**Figure 2:**
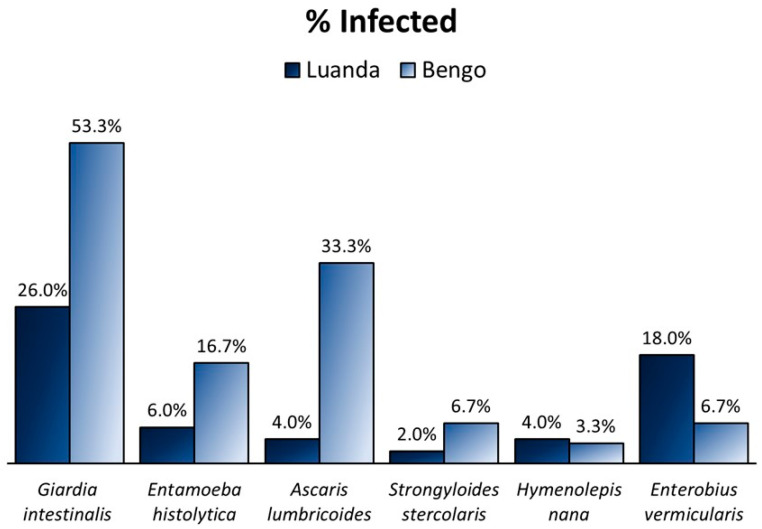
Prevalence of parasitic infections of individuals, namely controls and SCD before HU therapy (*n* = 80), from two different Angolan regions: Luanda (*n* = 50), the capital and largest city of the country, and Bengo (*n* = 30), a rural region, which is one of the provinces bordered by Luanda.

Considering the control and before HU groups, statistically significant differences between the prevalence of intestinal parasites were observed according to the place of residence (Figure 2, *n* = 80). The three highest differences in parasite prevalence, which are represented in Figure 2, were the following: 29.3% for *A. lumbricoides*, 27.3% for *G. intestinalis*, and 11.3% for *E. histolytica*. The statistical analysis revealed that children from Bengo (*n* = 50), compared to Luanda (*n* = 30), had a higher prevalence of *G. intestinalis* (*p* = 0.014) and *A. lumbricoides* (*p* = 0.001). Also, significant differences were found for the variable regarding being infected with at least one parasite (p = 0.003) and being co-infected with two or more parasites (*p* = 0.035), as in Bengo, 37% of children were coinfected, while in Luanda, only 16% presented more than one parasite. *Trichuris trichiura* was detected in only one child from the control group, who was also a resident from Bengo.

As for the SCD children after HU treatment (*n* = 33) from Luanda (*n* = 20) and Bengo (*n* = 13), the highest differences in parasite prevalences were: 33.5% for *G. intestinalis*, 30.8% for *A. lumbricoides*, and 23.1% for *E. histolytica*; all were higher in the Bengo residents. Additionally, there were no positive cases in Luanda in this group for the following parasites: *E. histolytica*, *A. lumbricoides*, *S. stercoralis*, and *H. nana*. Also, there were no positive cases in Bengo for *E. vermicularis*. The statistical analysis revealed significance and higher prevalence in the Bengo population for the identical variables of the previous group: *G. intestinalis* (*p* = 0.025) and *A. lumbricoides* (*p* = 0.017), infected (*p* < 0.001) and co-infected (*p* = 0.005). Although there was a notable difference in the prevalence of *E. histolytica*, this finding was not statistically significant (*p* = 0.052).

### 2.3. SCD Clinical Data and Parasitic Infections

**Figure 3:**
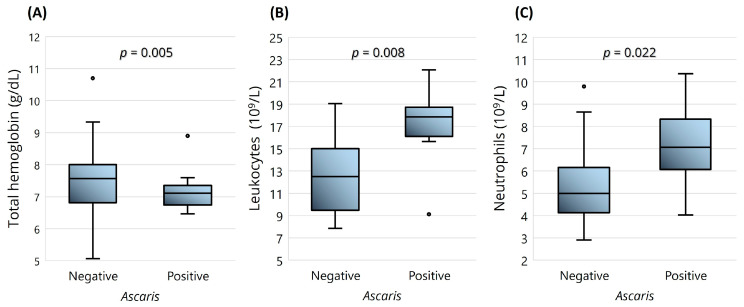
Boxplots representing the significant clinical differences in SCD children before HU treatment (*n* = 40), and infected with *A. lumbricoides* (*n* = 6). Hematological parameters: (**A**) total hemoglobin; (**B**) leukocytes; (**C**) neutrophils. The boxplots show the median, the first and third quartiles (bottom and top bars), the minimum and maximum values, and the outliers. Statistical significance was assessed using the Student’s *t*-test.

Regarding the hematological parameters and presence of parasites, the noteworthy results included the statistically significant differences in the levels of leukocytes (*p* = 0.008), neutrophils (*p* = 0.022), and hemoglobin (*p* = 0.005), with the first two higher and the latter lower in SCD children with ascariasis (Figure 3). The level of anemia (severe or moderate) and BMI group (normal or underweight) were also tested, but no statistically significant differences were found.

### 2.4. Impact of Parasites on Gut Microbiome

**Figure 4:**
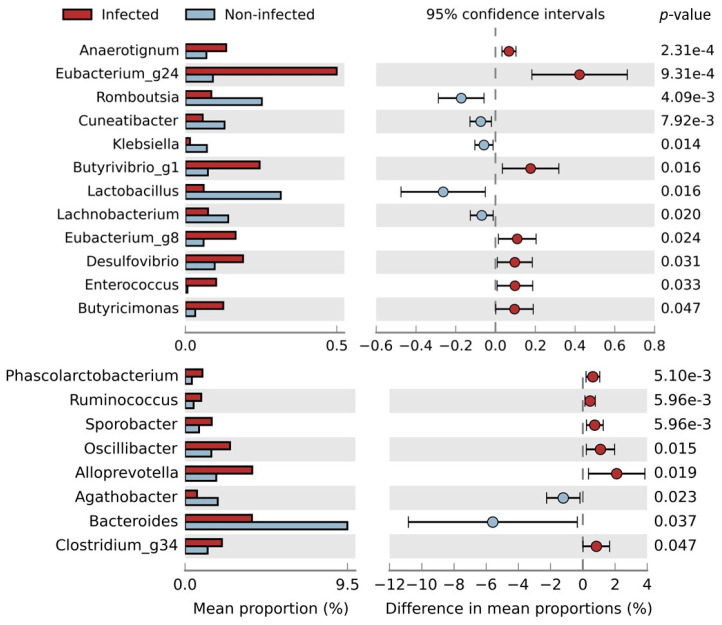
Distribution of the mean proportions of bacterial abundances between children infected with one or more parasites (*n* = 44) and those non-infected (*n* = 36) in a population of SCD children before HU treatment and in the controls (*n* = 80). The results were filtered employing a *p*-value lower than 0.05 using Welch’s *t*-test, the effect size with a 0.05 threshold, and by retaining unclassified reads in STAMP software (version 8.30).

The Angolan children with parasitic infections (*n* = 80) expressed higher numbers of the following bacteria (Figure 4): Anaerotignum, Eubacterium_g24, Butyvibrio_g1, Eubacterium_g8, Desulfovibrio, Enterococcus, Butyricimonas, Phascolarctobacterium, Ruminococcus, Sporobacter, Oscillibacter, Alloprevotella, and Clostridium_g34, whereas the non-infected children presented a higher abundance of Romboutsia, Cuneatibacter, Klebsiella, Lactobacillus, Lachnobacterium, Agathobacter, and Bacteroides. 

A similar statistical analysis of Figure 4 was also performed on the children before and after hydroxyurea (*n* = 73), but the only statistically significant results were obtained for *Lactobacillus*, which were higher (*p* = 0.008) in the non-infected, and *Oscillibacter*, which were higher (*p* = 0.015) in the infected children.

Looking more closely at how specific parasitic infections can impact certain bacteria (Table 1), it is notable that children infected with *G. intestinalis* exhibited lower numbers of *Bacteroides uniformis*, *Lactobacillus*, *Shuttleworthia*, and *Cuneatibacter* bacteria. As for those infected with *E. histolytica*, the *Lactobacillus*, *Bidifobacterium*, *B. uniformis*, *Roseburia*, and *Shuttleworthia* bacteria were diminished. The positive cases of *A. lumbricoides* showed lower numbers of *Bifidobacterium*, *Cuneatibacter*, *B. uniformis*, *Roseburia*, and *Shuttleworthia*. Finally, the positive cases for *E. vermicularis* had a decreased abundance of *Lactobacillus, Cuneatibacter*, and *Roseburia*. Moreover, *Roseburia intestinalis* was reduced in the group infected with *E. vermicularis* (*p* = 0.042) and *E. histolytica* (*p* = 0.025). Although *Bifidobacterium_uc* did not decrease significantly in *Enterobius*-positive children, a particular species of this bacteria, *Bifidobacterium bifidum*, was significantly reduced (*p* = 0.017). The *Bacteroides* genus was decreased in *Entamoeba*-positive (*p* < 0.001) and *Ascaris*-positive (*p* = 0.003) cases. *S. stercoralis*, *H. nana* and *T. trichiura* were excluded from this statistical analysis due to the low number of positives in the studied population.

## 3. Discussion

Parasitic infections are a common problem in developing countries and may contribute to the morbidity in SCD patients, increasing the severity of anemia and the need for transfusions [28]. Moreover, the intestine of SCD patients could be a primary entry point for several types of microorganisms due to its increased permeability caused by VOC episodes and its higher levels of activated neutrophils [8,23]. 

The SCD children infected with *A. lumbricoides*, one of the most prevalent helminths in the study population, showed that the parasite had a negative impact on the hematological parameters, i.e., higher values of leukocytes and neutrophils and lower mean hemoglobin. This is consistent with the results of other studies that have reported lower hemoglobin and hematocrit levels in SCD patients infected with intestinal helminths compared to those of noninfected subjects [29,30]. Both studies have also revealed higher leukocyte values in the infected group, although the difference was not significant. The lower hemoglobin level could be attributed to intestinal blood and nutritional deficiency [29], a common consequence of soil-transmitted helminths in the host intestine. *Ascaris lumbricoides*, as well as *T. trichuris* and *S. stercolaris*, are all classified as soil-transmitted helminths, since the transmission occurs through direct skin contact with contaminated soil, with infections typically occurring in areas with poor sanitation resources [31].

*Giardia intestinalis* was the most prevalent intestinal protozoan in the study population. Giardiasis is considered one of the most prevalent enteric diseases, affecting humans as well as animals, with infection rates ranging from 20 to 30% in low-middle-income countries [19,22]. The transmission of this protozoan occurs mostly through the oral–fecal route due to the ingestion of parasite cysts found in contaminated water or food [22]. Individuals with domestic or rural animals in their households have a two to five times higher risk of infection [32]. Furthermore, it is estimated that only 39% of rural Angolans have access to improved water sources and even fewer, 16%, have access to adequate sanitation [11]. All those factors may explain the higher parasite burden found in the rural area of Bengo compared to the children who live in the city of Luanda, especially for the *G. intestinalis* and *A. lumbricoides* parasites.

The slightly higher infection rates observed in the controls when compared to the SCD children was unexpected, since the latter are more prone to develop parasitic infections and present more severe symptoms due to their compromised immune systems. Although there are 3.5 billion people infected, only 450 million are seriously affected by parasites [10], since the immunocompetent individuals normally express mild or absent symptoms [22]. However, these SCD children frequently visit the hospital due to health complications and regular check-ups, where they are monitored by health professionals; therefore, a parasitic infection could be easily detected and treated during these consultations. 

Another interesting result is the fact that there was a reduction, although not statistically significant, of 44% in SCD Giardia-positives and of 76% in Enterobius-positives after 6 months of HU treatment. Hydroxyurea is a drug that blocks the cell cycle at the G1/S phase in other organisms by inhibiting DNA replication [33]. Recent studies have reported an antiparasitic activity of HU for some blood protozoans through the blockage of *Toxoplasma gondii* tachyzoites replication [34] or the synchronization of the kinetoplast DNA of several parasites, including *Leishmania* (*L. tarentolae*, *L. major*, *L. infantum*) and *Trypanosoma* (*T. brucei* and *T. cruzi*) [35,36]. Moreover, a clinical trial with SCD children reported that HU at the maximum tolerated dose is associated with lower malaria incidence [37]. HU can suppress the growth of *Plasmodium falciparum* and prevent cerebral malaria caused by *Plasmodium berghei* in mice [38]. However, there are some inconsistent results in the literature relating to intestinal parasites, with one study reporting significant effects on the cell cycle of *G. intestinalis* [39] and another reporting no inhibition of the growth of *Giardia* cultures [33]. The literature on this subject is rather scarce, and more studies are needed to determine the influence of HU on parasite replication cycles.

A study that analyzed the fecal microbiota composition of 1204 children from Guinea-Bissau reported a decreased abundance of *Lactobacillus* in protozoan-infected children and a lower number of *Bacteroides* in children infected with *E. histolytica/dispar* [17]. Our results are consistent with these findings, revealing a decreased number of *Lactobacillus* in the infected children for both the protozoans tested, *E. histolytica* and *G. intestinalis*. Another study stated that *E. histolytica*, through the selective phagocytosis of healthy bacteria (such as *Lactobacillus*), could be changing the bacterial gut microbiome toward a pathogenic profile [15]. Both *G. intestinalis* and *E. histolytica* are able to change the mucus composition, which leads to decreased bacterial attachment to gut epithelium [19]. Moreover, there are several *Lactobacillus* strains that have been shown to inhibit protozoan infections [16], i.e., by stimulating a humoral immune response against a parasite (e.g. *Giardia*) or by producing bacteriocins to inhibit the parasite adhesion [40]. Therefore, looking at the literature, two hypotheses are plausible: either the children’s baseline microbiota could be protecting them from developing a parasitic infection, or the presence of protozoans is reducing the number of beneficial bacteria through phagocytosis.

An interesting relationship between parasites and gut microbiota enterotypes was reported, describing that a type I enterotype (defined by *Bacteroides* spp.) was mainly associated with non-infected children, switching to a type II enterotype (determined by *Prevotella* spp.) upon infection with either the *G. intestinalis* protozoa or a mix of helminths such as *A. lumbricoides*, along with *T. trichiura* [14]. Our results are consistent with those in this study, as we observed that the infected children were associated with low levels of *Bacteroides*, particularly *Bacteroides uniformis*, a bacterium found in lower levels in the children infected with *G. intestinalis*, *A. lumbricoides*, and *E. histolytica*.

Several species of the genus *Bacteroides* and *Bifidobacterium* are considered beneficial, as they can produce short-chain fatty acids (SCFAs) in the host gut, which are important for homeostasis and immune modulation and can improve the intestinal mucosal barrier [40,41,42]. *Roseburia*, *Cuneatibacter*, and *Shuttleworthia* all belong to the *Lachnospiraceae* family, also recognized as main producers of SCFAs [43]. A study revealed that subjects infected with helminths (such as *A. lumbricoides, Necator americanus* and *T. trichiura*) were negatively related to the bacterial abundance of *Lactobacillus* and *Lachnospiracaea* [44], a finding also observed in the present study, both in the children infected with helminths and protozoans. Interestingly, *Clostriudium_g34*, a bacterium that we have demonstrated to be negatively correlated with HbF levels and positively correlated with two adhesion molecules (P-selectin and E-selectin) in the same SCD population [45], was more abundant in the infected children.

Understanding the processes and implications of parasite-microbiome interactions in the gut could lead to better or newer treatments for parasitic infections and gut modulation. Indeed, *Lactobacillus* and *Bifidobacterium* are already used as probiotics due to their ability to modulate the host immune system, the production of antimicrobial peptides, and the inhibition of pathogens [16]. Interventions targeting the microbiota, such as dietary interventions, probiotics, prebiotics, or symbiotics, hold great potential and could be introduced to attenuate immune dysregulation and gut dysbiosis, mitigating several inflammatory processes triggered by SCD.

## 4. Materials and Methods

### 4.1. Population and Sample Collection

Given that all participants were minors, written informed consent was obtained from the legal guardians of each subject. This process was carried out in accordance with an institutional review board-approved protocol to obtain clinical information, blood, and fecal specimens for research purposes, reviewed and approved by the Ethical Committee of the Ministry of Health of Angola and ESTeSL (parecer 07/2022 and CE-ESTeSL-Nº.43-2018). All patients underwent regular consultations with a pediatrician who collected the necessary clinical details. A total of 80 children were included in this study, of which 40 were SCD patients of Hospital Pediátrico David Bernardino or Hospital Geral do Bengo, and 40 were non-SCD patients (controls), who were siblings of these SCD patients. The first hospital is located in Luanda, the capital and largest city of Angola, and the second is in Caxito (Bengo Province), a rural area located 60 km away from Luanda. The SCD children were subjected to continuous hydroxyurea treatment (fixed dose of 20 mg/kg/day), and a total of 33 samples were collected after six months of intake, since seven patients did not attend the scheduled consultation. All 113 fecal samples were collected in DNA/RNA Shield Fecal Collection tubes (Zymo Research, Irvine, CA, USA) between the months of October 2020 and October 2021.

### 4.2. Clinical Analysis

Whole blood samples were collected from each SCD patient for hematological and biochemical analysis and electrophoresis for SCD confirmation. Hematological determinations were performed in an XT- 2000i Hematology Analyzer (Sysmex Corporation, Kobe, Japan), which included complete blood count (erythrocytes, white blood cells, reticulocytes, and platelets), hemoglobin, mean corpuscular volume (MCV) and mean corpuscular hemoglobin (MCH). The biochemical analysis using Cobas C111 (Roche Diagnostics, Basel, Switzerland) and Mindray BA- 88A (Mindray, Shenzhen, China) included the determination of lactate dehydrogenase (LDH), creatinine, urea, total and direct bilirubin, aspartate aminotransferase (AST), and alanine aminotransferase (ALT) levels.

### 4.3. Molecular Analysis

Gut microbial DNA was extracted from fecal samples using a ZymoBIOMICS™ DNA Miniprep Kit (Zymo Research, Irvine, CA, USA), according to the manufacturer’s instructions. The DNA samples were quantified and evaluated for purity using a NanoDrop One spectrophotometer (Thermo Fisher Scientific, Waltham, MA, USA), after which they were stored at −20 °C until further processing. The preparation of the libraries for 16S sequencing was performed with a Nextera XT DNA Library Preparation Kit in NextSeq 550 (Illumina, San Diego, CA, USA), as previously described [26,27]. The real-time PCR method was used for the detection of eight different parasites and was performed using the CFX Connect™ Real-Time PCR Detection System (Bio-Rad, Hercules, CA, USA), in a final volume of 20 μL. Real-time PCRs for *G. intestinalis*, *A. lumbricoides*, *S. mansoni*, *E. vermicularis*, and *T. trichiura* were performed using a mixture comprising NZYSupreme qPCR Probe Master Mix (NZYTech, Lisboa, Portugal), 0.3 μM of each primer, 0.1 μM of the probe, and approximately 5 ng/μL of DNA extract. The PCR thermocycling conditions were set at 95 °C for 5 min for polymerase activation, followed by 40 cycles of 95 °C for 5 s (denaturation) and 60 °C for 30 s (annealing and extension). For the real-time PCRs to detect *E. histolytica*, *H. nana*, and *S. stercoralis*, the NZYSupreme qPCR SYBR Green Master Mix (NZYTech, Lisboa, Portugal) was used with the same specifications mentioned for the probe PCR (Table 2). The only difference in the program was the addition of a melting-curve analysis conducted in the same thermocycler after the 40 cycles, which consisted of: 72 °C for 2 min, 60 °C for 5 s, and 95 °C, with increments 0.5 °C/s and a final step of 40 °C for 30 s, recording changes in fluorescence along with changes in temperature. Primer and probe oligonucleotide sequences, as well as melting point temperatures for the SYBR Green PCRs, are shown in Table 2. Each PCR round always included a DNA-positive control for a specific parasite, as well as a blank, consisting of a PCR mix without a template.

### 4.4. Statistical Analysis

All the statistical data calculations were performed using IBM SPSS Statistics version 27 software (IBM Corporation, NY, USA). Significant differences between groups for quantitative variables were analyzed using a Student’s *t*-test or a Mann–Whitney test. As for the categorical variables, the Chi-square test or the Fisher’s exact test was used for association tests. The results are presented as the mean ± standard deviation, and *p* < 0.01 was considered statistically significant.

## 5. Conclusions

This study opens new perspectives for understanding the interaction between parasites and the gut microbiome, presenting data on the prevalence of several protozoans and helminths as detected by molecular techniques in a high-transmission setting and where the information is scarce. Parasitic infections affect billions of people worldwide and can lead to tremendous disability and suffering, especially in children and immunocompromised patients, yet these can easily be controlled or eliminated using antiparasitic drugs. The SCD population is not only more susceptible to developing severe parasitic infections, but also faces greater challenges in combating infections, primarily due to its compromised immune systems. In developing countries, infections still remain a main cause of mortality in SCD patients, mainly due to a higher risk of exposure to pathogens and the presence of other co-morbidities such as malnutrition and diminished access to medical care [28]. All these factors provide a rationale for implementing important measures like improving personal hygiene practices, periodic stool examination, and regular deworming, with the objective of alleviating infection burden, avoiding worsening SCD symptoms, and ultimately improving the overall quality of life.

## Figures and Tables

**Table 1:** Statistical significance according to Welch’s *t*-test for infected SCD children and controls (*n* = 80) for six selected bacteria (*Lactobacillus*, *Bifidobacterium*, *Cuneatibacter*, *Bacteroides uniformis*, *Roseburia*, and *Shuttleworthia*), which showed a decreased abundance in positive cases of certain parasitic infections. Data were obtained using STAMP software with a filter for an effect size of a 0.01 threshold and a *p*-value lower than 0.05. Abbreviations: n.s. indicates “not significant”, and uc indicates “unclassified”, in terms of species.

	*Giardia* (+)	*Entamoeba* (+)	*Ascaris* (+)	*Enterobius* (+)
↓ *Lactobacillus_uc*	*p* = 0.010	*p* = 0.031	n.s.	*p* = 0.042
↓ *Bifidobacterium_uc*	n.s.	*p* < 0.001	*p* = 0.001	n.s.
↓ *Cuneatibacter*	*p* = 0.002	n.s.	*p* < 0.001	*p* = 0.029
↓ *Bacteroides uniformis*	*p* = 0.025	*p* = 0.017	*p* = 0.021	n.s.
↓ *Roseburia_uc*	n.s.	*p* = 0.026	*p* = 0.001	*p* = 0.040
↓ *Shuttleworthia_uc*	*p* = 0.011	*p* < 0.001	*p* = 0.003	n.s.

**Table 2:** Primers and probes for the detection of the following intestinal parasites by real-time PCR: *E. histolytica* (protozoa), *H. nana* (cestode), *S. stercoralis* (nematode), *A. lumbricoides* (nematode), *G. intestinalis* (protozoa), *S. mansoni* (trematode), *E. vermicularis* (nematode), and *T. trichiura* (nematode).

Target Parasite	Molecular Target	Oligonucleotides	Sequence (5′-3′)	Melting Temperature	References
***Entamoeba histolytica***	SSU rRNA	FW	ATTGTCGTGGCATCCTAACTCA	76.5 °C	[46]
		REV	GCGGACGGCTCATTATAACA		
***Hymenolepis nana***	ITS1	FW	CATTGTGTACCAAATTGATGATGAGTA	79.5 °C	[47]
		REV	CAACTGACAGCATGTTTCGATATG		
***Strongyloides stercoralis***	18S	FW	GAATTCCAAGTAAACGTAAGTCATTAGC	81.5 °C	[48]
		REV	TGCCTCTGGATATTGCTCAGTTC		
***Ascaris*** ***lumbricoides***	ITS1	FW	GTAATAGCAGTCGGCGGTTTCTT		[48]
		REV	GCCCAACATGCCACCTATTC		
		Probe	FAM-TTGGCGGACAATTGCATGCGAT-BHQ1		
***Giardia*** ***intestinalis***	SSU rRNA	FW	GACGGCTCAGGACAACGGTT		[46]
		REV	TTGCCAGCGGTGTCCG		
		Probe	FAM-CCCGCGGCGGTCCCTGCTAG-BHQ1		
***Schistosoma mansoni***	90 bp fragment of a highly repetitive 121 bp sequence of *S. mansoni*	FW	CCGACCAACCGTTCTATGA		[49]
		REV	CACGCTCTCGCAAATAATCTAAA		
		Probe	FAM-TCGTTGTATCTCCGAAACCACTGGACG-BHQ1		
***Enterobius*** ***vermicularis***	ITS1	FW	CGGTGTAATTTTGTTGGTGTCTATG		[47]
		REV	TGGCAGCATTGCAAACTAATG		
		Probe	FAM-TGTGCCAGTCAACGCCTAAACCGTC-BHQ1		
***Trichuris*** ***trichiura***	18S	FW	TTGAAACGACTTGCTCATCAACTT		[50]
		REV	CTGATTCTCCGTTAACCGTTGTC		
		Probe	FAM-CGATGGTACGCTACGTGCTTACCATGG-BHQ1		

## Data Availability

Data generated during this study are available from the corresponding author upon reasonable request.
